# The Microbiome as a Protagonist of Xylophagous Insects in Adaptation to Environmental Conditions and Climate Change

**DOI:** 10.3390/ijms262010143

**Published:** 2025-10-18

**Authors:** Alexander Kuprin, Vladislava Baklanova

**Affiliations:** Federal Scientific Center of the East Asia Terrestrial Biodiversity, Far East Branch of the Russian Academy of Sciences, Vladivostok 690022, Russia

**Keywords:** xylophagous insects, gut microbiota, wood decomposition, insect–microbe symbiosis, adaptive evolution, forest ecosystems, climate change, environmental stressors, insect ecology, omics technologies

## Abstract

Xylophagous insects represent a diverse group of species whose life cycles are trophically associated with wood at various stages of decomposition. In forest ecosystems, they play a pivotal role in wood degradation and biogeochemical nutrient cycling. Their remarkable adaptation to feeding on structurally complex and nutrient-poor woody substrates has been largely mediated by long-term symbiotic interactions with gut microbiota. This review synthesizes current knowledge on the molecular and ecological mechanisms underlying insect–microbiota interactions, with particular attention paid to the impact of environmental stressors—including elevated temperature, shifts in moisture regimes, and pollution—on microbial community structure and host adaptive responses. We critically evaluate the strength of evidence linking climate-driven microbiome shifts to functional consequences for the host and the ecosystem. The ecological implications of microbiota restructuring, such as impaired wood decomposition, decreased disease resistance, facilitation of xylophagous species spread, and alterations in key biotic interactions within forest biocenoses, are discussed. Particular emphasis is placed on the integration of multi-omics technologies and functional assays for a deeper, mechanistic understanding of microbiota roles. We also assess the potential and limitations of microbiome-based approaches for insect population management, with the overall goal of maintaining and enhancing the resilience of forest ecosystems under ongoing climate change.

## 1. Introduction

Xylophagous (wood-feeding) insects, acting as keystone decomposers, exert a major influence on the composition and dynamics of saproxylic (wood-associated) communities [1,2]. The decomposition of dead wood is a fundamental process that regulates the rate of carbon release, drives nutrient recycling, and creates microhabitats for a wide range of organisms [3,4,5]. These insects comprise a broad spectrum of taxa across multiple orders, including beetles (Coleoptera) [6,7,8], termites (Blattodea) [9], flies (Diptera) [10,11], and moths and butterflies (Lepidoptera) [12], among others, exhibiting diverse feeding strategies and interactions with host plants and substrates.

The ability of xylophagous insects to efficiently utilize wood as a nutritional resource is largely mediated by their symbiotic associations with a diverse gut microbiota (here used interchangeably with “microbiome” in an ecological context, while “microbiome” may also refer to the collective genetic material of these microbes) [13]. This microbiota constitutes a complex consortium of bacteria, archaea, fungi, and protists that jointly contribute to the breakdown of cellulose, hemicellulose, and lignin—the major but highly recalcitrant components of wood [13,14,15]. In addition, it provides other essential services, including atmospheric nitrogen fixation, biosynthesis of vitamins and amino acids, detoxification of plant secondary metabolites, and protection against pathogens [14,16,17]. Thus, gut microbiota represents an indispensable element of xylophagous insect physiology, enabling them to subsist on a diet composed almost exclusively of wood.

Forest ecosystems are experiencing unprecedented pressures linked to global environmental changes, particularly climate change [18,19,20]. These changes exert direct and indirect effects on xylophagous insect populations by altering wood availability and quality, reshaping microbiota structure and functionality, and modifying competitive and trophic interactions within saproxylic communities [14] and extreme climatic events such as droughts and wildfires are occurring with increasing frequency and intensity, causing widespread tree mortality and driving shifts in forest community composition [21]. Understanding the contribution of microbiota to the adaptation of xylophagous insects under these dynamic conditions is critical for predicting the future trajectories of forest ecosystems and for developing effective management strategies [22].

The aim of this review is to synthesize current knowledge on the role of microbiota in the adaptation of xylophagous insects to environmental and climatic changes. We highlight the molecular and ecological mechanisms that shape insect–microbiota interactions, analyze the impacts of climate change and other anthropogenic factors on microbiome structure and function, and discuss the potential applications of microbiome research for innovative strategies aimed at managing xylophagous insect populations and enhancing forest ecosystem resilience under global climate change.

This narrative review synthesizes current knowledge identified through searches in databases such as Scopus, Web of Science, and Google Scholar using key terms including “xylophagous insects”, “gut microbiota”, “wood decomposition”, “climate change”, and “symbiosis”, focusing on articles published between 2010 and 2025.

## 2. Diversity and Ecological–Physiological Characteristics of Xylophagous Insects

Xylophagous insects represent a highly diverse assemblage of organisms that play a crucial role in wood decomposition and nutrient cycling within forest ecosystems [1,2]. Comprehensive knowledge of their taxonomic classification, ecological functions, digestive system morphology, and microbiota composition forms a vital basis for advancing research on their adaptation to fluctuating environmental conditions.

### 2.1. Overview of Major Groups of Xylophagous Insects and Their Ecological Roles

The group of xylophagous insects includes diverse taxa that differ markedly in feeding strategies, anatomical specializations, and ecological impacts on forest ecosystems [23]. Below is a concise summary of principal groups, highlighting their key characteristics and ecological functions (see Table 1).

**Figure 1:**
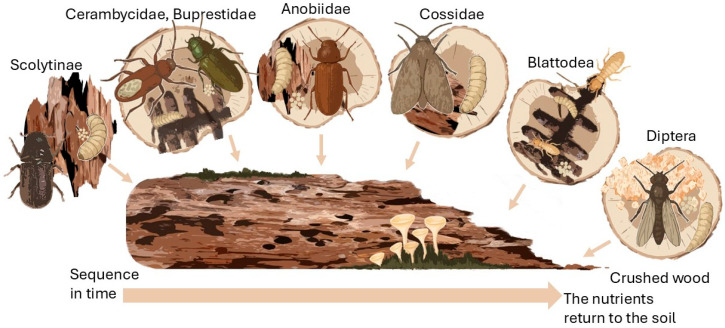
Succession of xylophagous insect communities during wood decomposition, illustrating the shift in dominant groups according to the stage of wood decay. This dynamic process is driven by changing substrate properties and microbial enrichment.

Wood decomposition proceeds as a non-random, sequential ecological succession marked by temporal and spatial heterogeneity within xylophagous communities. Throughout the stages of wood decay, dominant organismal groups successively replace each other in patterns that correspond to changes in the physicochemical properties of the substrate [3,23]. As presented in Figure 1, the temporal and spatial colonization pattern of wood by xylophagous insects exemplifies a successional model driven by progressive substrate transformation and microbial enrichment.

Early succession stages are commonly dominated by colonization of fresh or weakened wood by small bark beetles specializing in phloem feeding. These pioneers are succeeded by larger wood-boring beetles such as longhorn beetles (Cerambycidae) and jewel beetles (Buprestidae), capable of penetrating more lignified and drier wood. At advanced decay stages, furniture beetles (Anobiinae) and carpenter moths (Cossidae) establish, while termites possessing potent enzymatic capabilities alongside complex symbiotic gut communities, finalize the decomposition of wood enriched with fungal biomass. Ultimately, dipteran larvae consume residual fungal mycelia and extensively decayed substrates.

A thorough understanding of this successional framework and the distinct functional roles of different taxa is essential for evaluating the ecological contributions of xylophagous insects and anticipating their taxon-specific responses to environmental and climatic perturbations.

### 2.2. The Structure and Functions of the Digestive System of Xylophagous Insects: Adaptations to Wood Feeding

**Figure 2:**
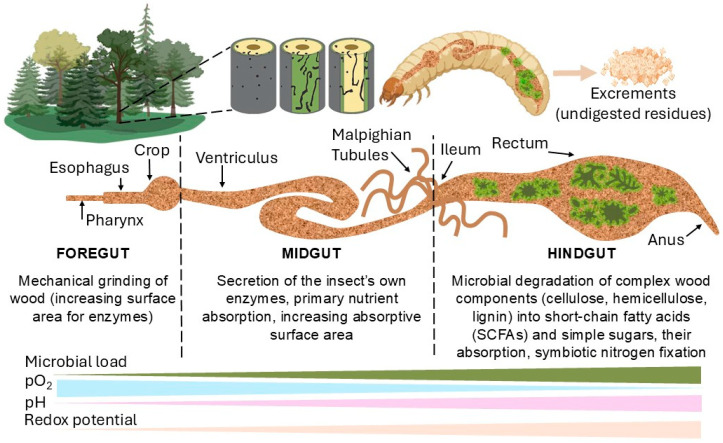
A simplified schematic representation of the digestive system of xylophagous insects illustrating key adaptations to wood feeding. The gut is divided into specialized compartments—foregut, midgut, and hindgut—each providing distinct physicochemical environments (e.g., pH, O_2_, redox potential, microbiota localization) optimized for different stages of wood digestion and nutrient absorption. Note: Actual gut anatomy and microbial niches vary significantly between taxa (e.g., termites vs. cerambycid beetles).

The digestive system of xylophagous insects exemplifies a remarkable evolutionary adaptation to a structurally complex and nutritionally deficient diet rich in cellulose, lignin, and other polysaccharides [36]. Crucially, gut morphology varies significantly across major taxa, reflecting differences in dietary habits and microbial symbiont composition [14]. A simplified diagram illustrating the generalized gut structure of xylophagous insects is shown in Figure 2, but it should be interpreted with an understanding of taxon-specific variations (e.g., the complex hindgut paunch in termites vs. the simpler systems in some beetle larvae).

The initial stage of wood digestion involves mechanical comminution. Many xylophagous insects, notably beetle larvae (e.g., Cerambycidae, Ptinidae: Anobiinae) and termites, possess robust mandibles capable of fragmenting and grinding wood into fine particles [37]. This mechanical processing significantly increases the surface area for degradation by insect cellulases/hemicellulases [36,38] and microbial lignocellulolytic enzymes [38].

The gut of xylophagous insects is highly compartmentalized, comprising specialized regions—the foregut, midgut, and hindgut—each providing optimal physicochemical conditions (e.g., pH, redox potential) for distinct phases of digestion and nutrient absorption and for hosting specific microbial communities [36,39,40]. In many xylophagous insects, particularly within the hindgut of termites and certain beetle larvae, anoxic (oxygen-deprived) conditions are established. Such environments are crucial for the activity of anaerobic microorganisms, which play an essential role in the degradation of cellulose and other recalcitrant polysaccharides [15]. The maintenance of these anoxic conditions is facilitated by multiple mechanisms, including the high metabolic oxygen consumption by resident microbes, specialized gut wall structures that restrict oxygen diffusion, and the synthesis of oxygen-binding compounds.

In conclusion, the digestive system of xylophagous insects constitutes a highly specialized and integrated complex evolved specifically for the degradation and assimilation of structurally resistant and nutrient-poor woody substrates. These adaptations, while serving a common function, exhibit significant taxon-specific variations that underpin distinct symbiotic strategies, allowing different xylophagous insects to occupy unique ecological niches and highlighting their pivotal role as bioreducers within the global carbon cycle.

## 3. Microorganisms and Their Role in Symbiosis with Xylophagous Insects

The interaction between xylophagous insects and their microbiota constitutes a complex symbiotic system in which bacteria, archaea, fungi, and protists collectively contribute to nutrition, immunity, and detoxification (see Table 2) [14]. Understanding the molecular mechanisms underlying these interactions, along with the modes of microbiota transmission and factors shaping its composition, is essential for assessing the role of microbiota in the adaptation of xylophagous insects to environmental and climate changes. The relative importance of these microbial functions can vary dramatically between insect taxa.

### 3.1. Role of Microbiota in the Digestion of Xylophagous Insects

The ability of xylophagous insects to efficiently exploit wood as a nutrient source largely results from their symbiotic associations with diverse gut microbiota [13]. Bacteria play a central role in digestion, particularly in cellulose degradation, the primary structural polysaccharide of wood. Cellulose is hydrolyzed by cellulolytic bacteria and fungi through enzyme complexes comprising endoglucanases, exoglucanases, and β-glucosidases that break it down into glucose, the main energy source for both the insect and its microbiota [41]. Although many xylophagous insects produce endogenous cellulases and hemicellulases [36,38], the principal contribution to lignocellulose digestion is often microbial [38] and can be taxon-dependent [42]. For instance, lower termites rely heavily on gut protists for cellulose breakdown [43], while higher termites and many beetles depend more on bacterial enzymes.

The dominant bacterial phyla commonly include Bacteroidetes, Firmicutes, and Proteobacteria; in termites, Spirochaetes are also prevalent [43,44]. Bark beetles harbor mainly bacteria from the order Enterobacterales, including family Erwiniaceae (e.g., *Pantoea*) and family Enterobacteriaceae (e.g., *Klebsiella*), as well as the family Bacillaceae (e.g., *Bacillus*), with some bacterial strains possessing strain-specific abilities to degrade resins and terpenes typical of coniferous trees [45,46,47]. In lower termites of the genus Reticulitermes, cellulolytic bacteria such as *Treponema* sp. and *Bacteroides* sp. maintain close symbiotic relationships with gut protists [48,49].

Hemicellulose is degraded by hemicellulases, primarily secreted by *Bacteroides* and other hemicellulolytic bacteria [50], yielding monosaccharides such as xylose, mannose, and galactose that serve as nutrients [15]. Given the low nitrogen content of wood, gut bacteria actively fix atmospheric nitrogen, converting N_2_ into ammonia, which is crucial for biosynthesis of amino acids and other nitrogenous compounds. Metatranscriptomic and metagenomic studies have revealed the active expression of nitrogen fixation genes (e.g., gene *nifH*) in gut microbiomes of various xylophagous insects [51,52,53], highlighting their key role in adaptation to nitrogen limitation. Diazotrophic communities in insect guts comprise genera from different families, such as *Klebsiella* (Enterobacteriaceae), *Pantoea* (Erwiniaceae), and termite-specific lineages [53].

Certain bacteria, including actinobacteria of the genus *Streptomyces*, produce lignin-degrading enzymes such as lignin peroxidases and laccases, facilitating lignin breakdown and thus improving access to other polysaccharides [54]. However, major lignin depolymerization often occurs in wood prior to ingestion, primarily mediated by fungi [55]. The products of lignin degradation support both the microbiota and host metabolism [15]. Additionally, *Enterobacter* sp. and *Pantoea* sp. synthesize B vitamins and essential amino acids, compensating for nutrient deficiencies in the woody diet and promoting insect growth [56,57].

Archaea, particularly methanogenic groups such as *Methanobrevibacter* and *Methanosphaera*, are widespread in the guts of xylophagous insects, especially termites [48,58]. They utilize hydrogen and carbon dioxide generated during cellulose fermentation to produce methane. This process removes hydrogen, maintaining optimal fermentation conditions by preventing inhibition of cellulolytic enzymes and detoxifying fermentation end-products, thereby reducing their toxicity to the host. Methanogenesis also enhances energy extraction efficiency from wood degradation [58].

Fungi play a major role in lignin degradation, a critical process for loosening wood structure [59]. Some yeasts and molds secrete enzymes that facilitate other microbes’ access to cellulose and hemicellulose [15]. Metagenomic studies have revealed a diverse arsenal of ligninolytic genes in wood-feeding systems [60]. Classic white-rot fungi such as *Trametes versicolor* and *Phanerochaete chrysosporium* produce lignin peroxidases, manganese peroxidases, and laccases, contributing to lignin mineralization [61,62]. Although these fungi are not consistently present in insect guts, their activity in wood aids insect feeding. Bark beetles, specifically, vector symbiotic ophiostomatoid (blue stain) fungi like *Ophiostoma* sp. and *Grosmannia* sp. [25,26], which include both mutualistic species aiding wood colonization and pathogenic species that weaken trees by modifying wood chemistry and suppressing competing microorganisms [63]. These fungi play a key role in facilitating beetle infestation and affecting host tree health [64].

Protists, particularly flagellates such as *Trichonympha* and *Pyrsonympha*, are specialized symbionts of lower termites [48]. They degrade cellulose efficiently using their own enzymes and intracellular bacteria [49,65]. These protists dominate digestion in lower termites [66], supplying bacteria with cellulose degradation products and establishing a complex gut trophic network [67].

### 3.2. Role of Gut Microbiota in Immune System Maintenance and Protection Against Pathogens

The gut microbiota of xylophagous insects plays an essential role in sustaining host immune function and defending against pathogens by balancing effective immune activation with prevention of excessive inflammation [68,69]. Gut microorganisms including *Bacillus*, *Lactobacillus*, and *Pseudomonas* continually stimulate the innate immune system by inducing expression of antimicrobial peptides (AMPs), e.g., defensins, cecropins, attacins, and diptericins, as well as defense molecules such as lysozymes in gut tissues and hemocytes [68,70]. Changes in microbiota composition can modulate AMP expression, enabling regulation of immune responses while minimizing chronic inflammation [71].

The microbiota also protects the host by competing with pathogens for nutrients and adhesion sites. Formation of biofilms by resident microbiota creates a physical barrier that hinders pathogen attachment to the gut epithelium, while nutrient competition limits pathogen growth and proliferation [72,73]. *Bacillus* and *Pseudomonas* produce bacteriocins and organic acids (for example, lactic, acetic, and propionic acids) that create inhospitable conditions for pathogens [74]. Additionally, gut microbiota supports intestinal homeostasis by stimulating mucin production, which forms a protective layer on the epithelial surface, limiting direct microbe–host contact and reducing inflammation [69,75]. Short-chain fatty acids (SCFAs) produced during fermentation also have immunomodulatory and antimicrobial effects [76].

Together, the gut microbiota functions as a complex immunomodulator and protective barrier that enhances resilience of xylophagous insects against pathogens. This role is particularly critical under climate change and anthropogenic stress conditions. Elucidating these mechanisms is fundamental for developing strategies to improve insect immunity, maintain host health, and control pest species prone to outbreaks that threaten forest ecosystems.

### 3.3. The Role of the Microbiota in the Detoxification of Harmful Wood-Derived Compounds

The microbiota of xylophagous insects plays a crucial role in detoxifying harmful compounds present in wood, including resins, terpenes, tannins, and lignin-derived phenolics, which can adversely affect insect digestion and immunity [77,78]. Specialized microorganisms enzymatically degrade and chemically modify each group of these secondary metabolites, thereby reducing the physiological burden on the host [79].

Resins and terpenes, particularly abundant in coniferous wood, are degraded by bacterial and fungal communities (e.g., *Pseudomonas*, *Sphingomonas*, *Trametes*) that produce oxidoreductases including microbial monooxygenases (including CYP-like enzymes), dehydrogenases, and epoxide hydrolases. These microbial enzymes often complement host detoxification pathways, such as host cytochrome P450s (CYPs), glutathione S-transferases (GSTs), and carboxylesterases (CCEs) [75,80,81,82]. These enzymes catalyze redox reactions and structural modifications (e.g., dealkylation, hydroxylation, acetylation), converting terpene compounds like α-pinene into less toxic and more soluble derivatives like α-terpineol and limonene, thereby facilitating detoxification and elimination [79,83].

Phenolic compounds—including tannins and lignin-derived phenolics—typical of deciduous wood, are broken down by bacteria and fungi (e.g., *Phanerochaete* sp., *Pleurotus* sp.) that secrete peroxidases, manganese peroxidases, and laccases [75,84]. These enzymes oxidize phenolics, forming radicals that polymerize into less harmful products or are reduced, resulting in decreased toxicity, improved digestion, and enhanced insect resistance [79].

Microbiota-mediated detoxification alleviates immune stress in the host, enabling more effective defense against pathogens and environmental stresses. Conversely, the insect’s immune system regulates microbial community composition to maintain gut homeostasis [14]. Additionally, some gut bacteria produce antioxidant enzymes, such as superoxide dismutase and catalase, which protect host cells from oxidative damage induced by wood-derived toxins [85]. Understanding these intricate interactions may inform novel strategies to protect xylophagous insects from the harmful effects of wood toxins, particularly under climate change and increasing environmental pollution.

**Table 2:** Functions of xylophagous insect microbiota, molecular mechanisms, and their role in adaptation.

Function of Microbiota	Molecular Mechanisms	Example Genera of Microorganisms	Key Enzymes/Molecules	Notes/Role in Adaptation	Sources
Cellulose degradation	Hydrolysis of cellulose to glucose	Bacteroidetes, Firmicutes, and Proteobacteria (bacteria), *Trichonympha* and *Pyrsonympha* (protists)	Cellulases (endoglucanases, exoglucanases, β-glucosidases)	Provides a basic energy source for the host and microbiota	[43,44,48]
Hemicellulose degradation	Hydrolysis of hemicellulose to sugars	*Bacteroides* (bacteria)	Hemicellulases	Allows use of additional carbohydrate sources	[50]
Lignin degradation	Oxidation of lignin polymers	*Streptomyces* (bacteria), *Phanerochaete* (fungi)	Lignin peroxidases, manganese peroxidases, laccases (primarily fungal, often acting in wood)	Reduces wood toxicity, improves access to cellulose and hemicellulose	[54]
Redox balance in the gut	Utilization of hydrogen and CO_2_ produced during cellulose fermentation	*Methanobrevibacter* and *Methanosphaera* (archaea)	Methanogenesis (e.g., methyl-CoM reductase)	Maintains optimal fermentation conditions, prevents accumulation of toxic products	[48,58]
Atmospheric nitrogen fixation	Conversion of N_2_ into ammonia	*Klebsiella*, *Pantoea*, (bacteria)	Nitrogenase (e.g., protein NifH)	Provides nitrogen for synthesis of amino acids and proteins	[52,53]
Synthesis of vitamins and amino acids	Biosynthesis of B-group vitamins and essential amino acids	*Enterobacter* sp. and *Pantoea* sp. (bacteria)	Vitamin and amino acid-synthesizing enzymes	Compensates for nutrient deficiency in wood, promotes insect growth and development	[57]
Stimulation of immune response	Induction of antimicrobial peptide expression	Resident microbiota	Antimicrobial peptides	Maintains immune homeostasis, prevents pathogenic inflammation	[68]
Competitive exclusion of pathogens	Competition for nutrients and binding sites	Resident microbiota	-	Prevents colonization by pathogens, limits growth of pathogenic microorganisms	[72,73]
Production of antimicrobial substances	Synthesis of bacteriocins and organic acids	*Bacillus*, *Pseudomonas* (bacteria)	Bacteriocins and organic acids	Actively suppresses growth and colonization of pathogens	[74]
Immunomodulation and maintenance of gut homeostasis	Support of gut homeostasis, production of mucin and short-chain fatty acids	Resident microbiota	Mucin, short-chain fatty acids	Reduces inflammation, immunomodulation	[69,75,76]
Resin and terpene metabolism	Oxidation and modification of toxic compounds	*Pseudomonas*, *Sphingomonas* (bacteria), *Trametes* (fungi)	Oxidoreductases, laccases	Reduces toxicity of wood resins and terpenes, facilitates wood digestion	[75,79,80,81,82]
Phenol degradation	Oxidation of phenolic compounds	*Phanerochaete*, *Pleurotus* (fungi)	Lignin peroxidase, manganese peroxidase	Eliminates toxic phenols, improves habitat and digestion	[75,84]

Note: Functions may be performed by gut microbiota or by microorganisms in wood substrate prior to or during insect feeding.

Together, the molecular dialogs between microbiota and host provide comprehensive digestive support, robust immune protection, and efficient detoxification of harmful compounds, collectively enabling xylophagous insects to adapt successfully to the challenges posed by a lignocellulosic diet and a changing environment.

### 3.4. Transmission Pathways of the Microbiota and Factors Influencing Its Assembly

**Figure 3:**
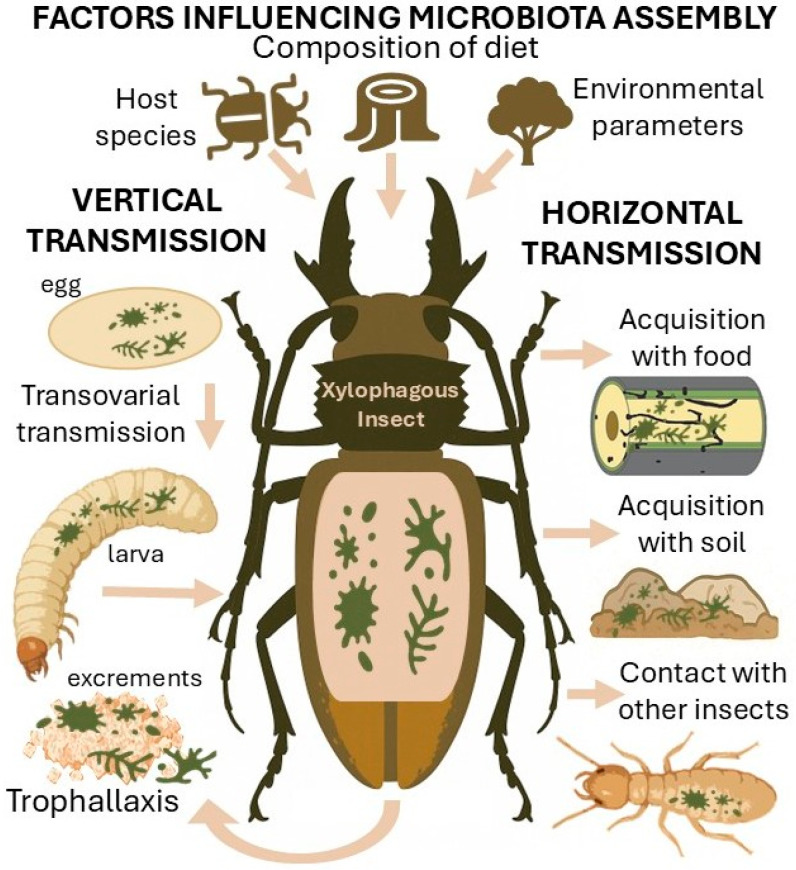
A simplified diagram of the microbiota transmission pathways in xylophagous insects (vertical and horizontal) and the main factors influencing its composition: host species, type of diet, environmental parameters (temperature, humidity).

The microbiota of xylophagous insects is transmitted primarily through two pathways: vertical (parent to offspring) and horizontal transmission (from the environment) (Figure 3) [86]. These pathways ensure both the stability of essential symbionts and the potential for adaptation to changing environmental conditions [87].

Vertical transmission involves the transfer of key symbiotic microorganisms from parents to offspring. This can occur via several mechanisms: transovarial transmission, where microbes are incorporated inside the oocytes, or transovum transmission, where they are smeared on the egg surface [86]. Other routes include inoculation through fecal matter [88] and social behaviors such as trophallaxis. In termites, this involves both proctodeal (anal feeding) and stomodeal (oral feeding) exchange of fluids [89]. Microbes can also be acquired through secretions from glands, for example, from salivary or exocrine glands in some wood-boring beetles [90,91]. This transmission route promotes microbiota stability across generations and can be associated with adaptation to specific wood types [92]. However, it may also limit microbial diversity and constrain the microbiota’s capacity to adapt to novel environmental challenges.

Horizontal transmission occurs by acquiring microbiota from the external environment [86], including food sources (wood colonized by microorganisms [60,61]), soil, gallery and frass contamination [93], and contact with other insects [25]. Bark and ambrosia beetles possess specialized fungal transport organs called mycangia, which carry fungal propagules acquired primarily from galleries rather than through direct insect-to-insect contact [93]. Additionally, horizontal transmission can occur via phoretic spores attached to beetle exoskeletons [25]. These mechanisms enhance microbiota diversity and facilitate more rapid adaptation to new ecological conditions and dietary substrates [94].

The assembly of the microbiota is further influenced by the insect species itself, the composition of its diet, and environmental parameters such as temperature and humidity [95,96,97,98]. However, since host identity and diet composition are often confounded in studies, disentangling their individual effects remains challenging [73]. Some studies have included appropriate controls to separate these factors, but caution is warranted in attributing microbiota composition changes to any single factor [99]. The interplay of these variables collectively shapes the unique microbial community structure and functional profile within the gut of each xylophagous insect species.

Consequently, the coevolutionary relationship between xylophagous insects and their microbiota has led to symbiotic microorganisms that frequently cannot survive outside the host. Simultaneously, the survival and success of these insects are critically dependent on their gut microbiota. The “xylophagous insect–microbiome” system undergoes continual modification under the influence of extreme environmental stressors and climate change, enabling host adaptation both throughout ontogeny and in varying ecological contexts. Notably, microbiome function depends not only on the internal physicochemical milieu (endoecology) but also on the external ecological environment, the habitat occupied by the macroorganism host.

## 4. Impact of Environmental and Climate Changes on Microbiota and Adaptation of Xylophagous Insects

Global climate and environmental changes can significantly influence the microbiota of xylophagous insects, altering their capacity for wood degradation and survival. Key factors considered include temperature rise, humidity fluctuations, and pollution [100]. These factors elicit shifts in microbiota composition and functionality, though effects may be direct on microbial communities or indirect via changes in the host physiology or wood substrate [101]. It is important to note that while several studies report notable changes, the magnitude and mechanisms of these effects vary depending on ecological context, insect taxon, and the methodological approaches used, requiring further quantitative and experimental investigation [102,103]. This section explores how these factors affect the xylophagous insect microbiota and their adaptive responses to changing conditions.

### 4.1. Impact of Rising Temperatures on the Microbiota and Adaptation of Xylophagous Insects

Temperature elevations driven by climate change exert both direct and indirect influences on the microbiota of xylophagous insects [104]. Observational and limited experimental studies suggest that increased temperatures can shift community composition toward heat-tolerant taxa and reduce the diversity of mesophilic microorganisms [102], with temperature ranges typically spanning from moderate (20–30 °C) to high (>30 °C) bands depending on the insect habitat and geographic region. These shifts may potentially impair wood degradation efficiency and alter immune markers in some taxa. However, causal evidence from manipulative experiments within relevant insect systems is often lacking, and many claims are extrapolated from microbial ecology principles or soil studies.

Additionally, elevated temperatures also alter tree physiology, increasing vulnerability to pests and diseases, and can modify wood chemical profiles, including resin and terpene levels, which are species- and stress-dependent [105,106]. For example, a slight increase in temperature has been shown to double terpene concentrations in non-mature needles of Norway spruce (*Picea abies*) and to increase sesquiterpene levels in Scots pine (*Pinus sylvestris*) [107,108]. These changes can adversely affect microbiota viability and enzymatic activities [109]. Furthermore, thermal stress modifies interactions among xylophagous insects, fungi, and nematodes, further impacting microbiota structure [110,111].

Certain xylophagous insects may adapt to higher temperatures through shifts in their microbiota composition, acquiring microorganisms optimized for functioning under altered conditions [112]. Adaptation here refers to measurable changes in host performance or fitness, such as increased survival, growth rate, or enzyme efficiency under thermal stress. For example, studies on *Aedes aegypti* have shown shifts toward heat-tolerant taxa like *Bacillus*, correlated with altered thermal tolerance and life history traits [102]. However, more taxon-specific case studies and experimental evolution approaches are needed to fully characterize these adaptive responses in xylophagous insects and establish causality. Horizontal gene transfer among microbes may facilitate rapid trait gain in microbial communities [113]. Understanding these mechanisms is for developing strategies—such as selecting for microbial communities that confer thermal tolerance—to potentially improve the capacity of xylophagous insects to withstand climate change [114]. These assumptions remain not fully explored and require more detailed investigations ranging from laboratory to field studies.

### 4.2. Impact of Humidity Changes (Droughts, Floods) on Microbiota and Adaptation of Xylophagous Insects

Fluctuations in humidity, particularly droughts and floods, impose significant hydric stress on xylophagous insects and their microbiota, affecting microbial composition (α- and β-diversity) and function, such as enzymatic activity and immune marker expression [115,116]. However, direct experimental evidence linking these changes to causal mechanisms and fitness outcomes in most xylophagous insects remains limited, with many inferences drawn from observed correlations or studies in other insect groups.

Drought conditions can weaken tree defenses, increasing their susceptibility to colonization by bark beetles and other xylophages [117]. Concurrently, drought alters wood chemistry—often increasing sugar content while reducing overall nutritional quality—which can indirectly shape the insect gut microbiome, though the specific metabolic consequences for the insect host are not fully quantified [117]. Observational studies suggest that dryness may promote the proliferation of opportunistic or pathobiont taxa within the gut, potentially correlating with reduced insect immunity and survival rates [118,119]. Furthermore, drought-induced modifications of nest and gallery microclimates (e.g., in termite mounds or beetle tunnels) are hypothesized to impede the activity of sensitive digestive microbes [116,120]. The anoxic conditions of the insect gut may be further exacerbated by drought, driving compositional shifts toward anaerobic taxa, but causal evidence from in vivo studies is needed to confirm this [121].

Conversely, flood events cause significant mortality in xylophagous insects and their associated microbiota, particularly for soil- and wood-dwelling species like termites and some beetle larvae [121]. A primary study on termites demonstrated a measurable loss of gut cellulolytic activity following flooding, directly linking the environmental stressor to a key microbial function [115]. Floods also facilitate the dissemination and proliferation of generalist entomopathogenic fungi, such as *Metarhizium*, which can exploit stressed insect hosts [122]. The processes and timescales of microbiota recovery following such disturbances are a critical area for future research.

In response to humidity fluctuations, many xylophagous insects exhibit behavioral and physiological adaptations, such as adjusting life cycle timing, selecting microhabitats with stable humidity, and engineering nest or gallery structures to buffer against external moisture variability [3,117,123]. There is emerging, though largely correlative, evidence that insects may selectively acquire microbial partners better suited to stress conditions via increased horizontal transmission [94,113,118]. For instance, under drought stress, some bark beetles appear to associate with more xerotolerant fungal strains from their environment [124,125]. This proposed mechanism could ensure the continuity of digestive and detoxification functions, but experimental validation is required to distinguish active selection from passive enrichment.

### 4.3. Impact of Environmental Pollution (Heavy Metals, Pesticides) on Microbiota and Adaptation of Xylophagous Insects

Environmental pollution, notably from heavy metals and pesticides, presents a potent anthropogenic stressor that can disrupt the composition and function of xylophagous insect microbiota [126]. The evidence for toxic effects is growing, but the field is characterized by in vitro findings and correlative field observations, with a scarcity of manipulative experiments that establish causality within the insect holobiont context.

Heavy metals (e.g., Pb, Cd, Cu, Zn) can accumulate in insect tissues and are known to inhibit microbial enzymes, induce oxidative stress, and cause cellular damage [127,128]. Exposure often leads to a reduction in overall microbial diversity and selects for metal-resistant bacterial taxa, as observed in field studies of insects in contaminated areas [129]. In vitro studies have identified specific bacterial genera, such as *Pseudomonas*, with the capacity to bioaccumulate or transform metals, suggesting a potential for bioremediation [130,131]. However, the in vivo protective role of these microbes for their insect hosts against metal toxicity remains largely hypothetical and requires direct testing.

Pesticides used in forest management can have profound, non-target effects on insect symbionts. Insecticides can reduce populations of beneficial gut bacteria essential for nitrogen fixation and detoxification, thereby disrupting these critical symbiotic services and potentially diminishing host fitness [132,133,134]. Furthermore, certain pesticides are known to suppress insect immune responses, creating a deleterious feedback loop where microbiota disruption and host immunocompetence decline synergistically [135]. While these patterns are supported by multiple studies, the precise cause-and-effect relationships and their variation across different insect-taxon and pesticide combinations are not fully resolved.

Some microorganisms associated with insects possess validated in vitro capacities for pesticide degradation or metal detoxification [134,136]. This has generated interest in the potential for microbes to mitigate pollution impacts on their hosts [133]. Nevertheless, translating these findings into practical applications, such as using probiotic consortia to enhance insect resilience in polluted forests, remains highly speculative. Significant challenges, including the stability of introduced microbes, ecological risks, and the complexity of host-microbe-pollutant interactions in natural settings, must be rigorously addressed through phased experimental frameworks before such strategies can be considered viable.

### 4.4. Adaptive Potential and Limitations of Xylophagous Insect Microbiome in Supporting Forest Ecosystem Resilience Against Climate Change

Forest ecosystem resilience to climate change critically depends on the adaptive capabilities of constituent organisms, including xylophagous insects and their microbiomes [101,112,137]. The xylophagous insect microbiome plays a pivotal role in sustaining ecosystem functions that uphold forest health [137], yet its potential and inherent constraints must be accounted for in ecosystem management.

The microbiome’s adaptive potential is driven by mechanisms such as horizontal gene transfer, facilitating rapid genetic innovation. This can theoretically enable new metabolic capabilities, such as the degradation of modified wood substrates or the detoxification of climate-induced plant compounds, potentially enhancing host fitness and performance [138,139,140]. Climatic stressors impose selective pressures that favor well-adapted microbial taxa. This selection fosters evolutionary flexibility, leading to a coordinated response where the adapted microbiome contributes to the host’s overall resilience to environmental shifts [102,112]. Furthermore, microbiome plays a crucial role in modulating insect immunity. For instance, in certain termite species, gut bacteria have been shown to upregulate the production of antimicrobial peptides (AMPs), potentially enhancing the host’s resistance against pathogens that may proliferate under climate change [68,69,141].

Nonetheless, limitations exist: the intimate interdependence of microbiome, insect physiology, and host genetics implies that without concurrent host adaptation, the microbiome alone cannot fully offset adverse environmental impacts [44,142,143]. Limited genetic diversity within the microbiome can restrict its evolutionary plasticity, and the complexity of ecosystem interactions complicates precise predictions regarding microbiome and host population responses to climate change [112]. The mode of microbiota transmission (vertical vs. horizontal) also fundamentally shapes adaptive potential, with vertical transmission promoting stability but potentially limiting novelty, and horizontal transmission enhancing diversity but introducing variability.

Therefore, a thorough understanding of both the adaptive capabilities and limitations of xylophagous insect microbiomes is essential for bolstering forest resilience and formulating integrated climate adaptation strategies. Future research should focus on testing the causality and strength of these adaptive mechanisms through controlled experiments.

## 5. Ecological Consequences of Changes in Xylophagous Insect Microbiota Under Climate Change

Climate- and anthropogenic-driven changes in the microbiota of xylophagous insects produce profound cascading effects on forest ecosystems, disrupting wood decomposition dynamics, reducing resilience to diseases and pests, and facilitating the spread of invasive insect species.

### 5.1. Disruption of Wood Decomposition and Nutrient Cycling

Alterations in microbiota diminish the efficiency of cellulose, lignin, and hemicellulose degradation, thereby slowing wood decomposition and nutrient input into soil, negatively impacting soil fertility, plant growth, and soil community structure. For example, elevated temperatures and drought conditions reduce cellulolytic bacterial diversity in termite guts, impairing deadwood decomposition and carbon cycling [115]. Accumulated lignin and toxic terpenes may inhibit decomposer microorganisms, disturbing soil microbial balance [144].

### 5.2. Spread of Invasive Species

Climate change facilitates the proliferation of invasive xylophagous insects harboring adapted microbiota that enable efficient exploitation of food resources and competition with native species [31,145]. Such invasive species may also vector pathogens responsible for mass tree mortality and alter community composition, resulting in reduced biodiversity and ecosystem resilience. For instance, the spread of invasive bark beetles (e.g., some *Dendroctonus* spp.) and ambrosia beetles (e.g., *Euwallacea* spp.) is often linked to their specific fungal symbionts, which can be more virulent or competitive in new environments [91,146]. A prominent example is the invasive bark beetle *Xylosandrus crassiusculus*, whose symbiotic fungus *Ambrosiella roeperi* contributes to its successful establishment in non-native forests [147,148]. The role of symbionts in facilitating host shifts and invasion success is further supported by studies on other ambrosia beetles and bark beetles [149,150,151].

### 5.3. Alteration of Key Biotic Interactions

The microbiota of xylophagous insects modulates interactions with fungi, bacteria, and other insects [141,152]. Restructuring of these microbial communities can affect fungal transmission and colonization, influence nitrogen fixation and detoxification processes, and impact the abundance of predators and competitors within the community. These biotic interactions directly influence forest ecosystem functioning and resilience, as biodiversity underpins essential ecosystem services [153].

Table 3 summarizes the ecological consequences of environmental stressor-induced microbiota changes in xylophagous insects, emphasizing their effects on insect adaptation and forest ecosystem resilience.

Changes in xylophagous insect microbiota under environmental stressors exert multiplicative negative effects on forest ecosystems, leading to diminished resilience and biodiversity. Effective forest conservation and management demand an integrated approach that incorporates microbiological processes, biotic interactions, and impacts of global climate change.

## 6. Future Research Directions and Practical Perspectives

Future research on xylophagous insect microbiota in the context of climate change should prioritize elucidating the specific molecular mechanisms underpinning insect–microbiota interactions, alongside their symbiotic, evolutionary, and adaptive relationships [154,155]. This necessitates integrating advanced omics technologies—including metagenomics, metatranscriptomics, proteomics, and metabolomics [156]—with robust functional validation and critical appraisal of methodological limitations.

### 6.1. Methodological Considerations and Integrative Approaches

Genomic analyses identify key enzymes and adaptive genes [38], metatranscriptomics reveal gene expression responses to climate shifts [157], proteomics provide profiles of active proteins [158], and metabolomics determine pivotal metabolites and their effects on insect physiology [159]. However, each method has inherent limitations. Amplicon sequencing (e.g., 16S rRNA) suffers from primer bias and low taxonomic resolution [160]. Shotgun metagenomics can reveal functional potential but often lacks linkage to specific taxa without advanced binning, which is challenging for complex communities [161]. Metatranscriptomics indicates expression but not necessarily protein activity or final metabolic flux [162]. Therefore, future work must employ integrative pipelines that combine omics with cultivation-based techniques, stable isotope probing (SIP), enzyme assays, and gnotobiotic models to establish causative links between microbiome composition, function, and host phenotype [94,163].

### 6.2. Hypothesis-Driven Mechanistic Research

Beyond descriptive studies, research should test explicit hypotheses derived from eco-evolutionary theory [164,165]. Xylophagy and saproxylophagy represent an example of symbiotic interactions, but our understanding of how they evolved remains fragmented across different disciplines, which prevents a comprehensive synthesis of insights. Recent advances in sequencing technology, bioinformatics, and conceptual frameworks like the holobiont theory provide new opportunities for integrating the fields of insect phylogenetics, morphology, enzymology, physiology, and microbial ecology [166,167]. Such an integrated approach, together with comparative data on the biology and functional ecology of saproxylic insects, opens promising opportunities for studying the origins of xylophagy and saproxylophagy, with an emphasis on the complex interactions among morphological, physiological, microbial, and ecological factors [13].

### 6.3. Practical Applications and Associated Challenges

Practically, insights from rigorous research could enable the use of microbiota as sensitive biomarkers for forest biomonitoring and early detection of adverse climate- or pollution-related ecosystem changes [129,168]. The development of effective microbiota management strategies, such as using probiotics or prebiotics, microbiota transplantation, or habitat manipulation, aims to bolster insect resilience to extreme temperatures, drought, and pathogens. However, these approaches are currently highly speculative and face significant challenges.

Probiotics and prebiotics have shown promise in controlled laboratory settings to improve digestion, immunity, and detoxification efficiency in some insect models, potentially contributing to better host growth and fitness. Microbiota transplantation could theoretically help restore microbial balance following environmental stress [169]. Importantly, these interventions are designed to avoid unintended negative impacts on insect physiology or ecosystem balance. However, moving these concepts to forest management requires careful consideration of ecological risks (non-target effects, unintended consequences for native insect communities, potential for horizontal gene transfer of undesirable traits (e.g., antibiotic resistance), and the risk of facilitating pathogen spread), technical feasibility (scalability, cost-effectiveness, and methods for delivery and establishment of microbial consortia in complex forest environments and within insect hosts), regulatory hurdles (approval processes for releasing modified or non-native microorganisms into the environment).

Management suggestions should be reframed as long-term research pathways rather than immediate solutions. A phased experimental framework—from laboratory proof-of-concept using gnotobiotic insects to mesocosm studies simulating forest conditions and small-scale, tightly monitored field trials—is essential to assess efficacy, risks, and feasibility [157]. Monitoring should include not only the target insect and its microbiome but also broader ecosystem impacts.

This comprehensive and cautious strategy will facilitate the establishment of scientifically sound biodiversity monitoring protocols, identification of promising microorganisms for biopreparations, and fostering conducive conditions for beneficial microbiota development through sustainable forest management. These initiatives will aid restoration of rare and endangered xylophagous insect populations and enable effective control of outbreaking forest and agricultural pests. Ultimately, such integrated, grounded in robust mechanistic understanding and careful risk assessment, may contribute to adaptive forest management, biodiversity conservation, and enhanced ecosystem resilience under climate change.

The following table (Table 4) summarizes key findings from hypothesis-driven primary studies on specific insect systems and juxtaposes them with identified gaps, proposing targeted experimental approaches to move the field from correlation to causation and towards predictive understanding.

## 7. Conclusions

Xylophagous insects constitute a diverse assemblage spanning multiple insect orders, characterized by significant differences in gut anatomy, symbiotic partnerships, and microbiota transmission modes. Their extensive diversity and successful adaptation to low-protein diets have been shaped by long-term, taxon-specific coevolution with symbiotic microorganisms, leading to the establishment of intricate and varied digestive associations. These microbial partners produce metabolites essential for the degradation of complex carbohydrates, facilitate the transformation of nitrogenous compounds, and support the insects’ adaptation to environmental challenges, thereby contributing to their evolutionary success.

Diet is the principal determinant of the diversity and composition of the gut microbiome in xylophagous insects. Consequently, future research should prioritize the investigation of the native microbiota of xylophagous insects using advanced omics approaches, high-throughput sequencing technologies, and cutting-edge microscopy techniques. Such integrative methodologies will enable a deeper understanding of the molecular mechanisms underpinning insect–microbiota symbioses and will inform the development of effective strategies for pest population management as well as the conservation of rare and threatened species. At a broader ecological level, the implementation of these carefully validated approaches may enhance the resilience of terrestrial ecosystems through the preservation of vital biodiversity components and a nuanced understanding of the complex interactions between insects, their microbes, and a changing world.

## Figures and Tables

**Table 1:** Key groups of saproxylic insects, their feeding characteristics, ecological roles, and representative families/genera.

Group	Taxa	Feeding and Characteristics	Ecological Role	Sources
Coleoptera	Curculionidae: Scolytinae, Cerambycidae, Ptinidae: Anobiinae, Buprestidae	Various stages of wood: phloem, sapwood, dense wood. Larvae create tunnels in wood; adults sometimes feed on leaves or bark.	Decomposition of dead and weakened wood, creating habitats for other organisms, influence carbon cycle; some species are forest and structural pests.	[6,7,8,24,25,26,27,28,29,30,31]
Blattodea	Various genera within Blattodea	Social insects; feed on dry or decayed wood, soil detritus. Build large nests.	Efficient wood decomposition, improving soil structure, bio-enrichment, important in food chains.	[9,32,33,34]
Diptera	Mycetophilidae, Syrphidae, Stratiomyidae	Larvae feed on fungal mycelium and decomposing organic matter.	Additional decomposition of wood and organic matter, participation in trophic networks, often underestimated wood decomposers.	[10,11,35]
Lepidoptera	Cossidae	Specialized larvae bore deep tunnels inside trunks and branches of living and dead trees.	Creation of cavities and acceleration of wood decomposition, weakening trees and increasing susceptibility to diseases.	[12]

Note: The table includes primarily xylophagous taxa as well as some saproxylic and fungivorous species that contribute to wood decomposition processes.

**Table 3:** Effects of environmental factors on microbiota of xylophagous insects, their adaptation, and consequences for forest ecosystems.

Environmental Factor	Effects on Xylophagous Insect Microbiota	Impact on Insect Adaptation and Condition	Consequences for Forest Ecosystems
Temperature rise	Shift in dominant taxa, increase in thermophiles, decrease in microbial diversity; changes in enzymatic functions	Reduced enzymatic activity, immune suppression	Slowed wood decomposition, accumulation of dead organic matter, decreased biodiversity
Drought	Proliferation of pathogens, microbial community imbalance, weakening of symbionts	Increased susceptibility to infections, reduced viability	Pest outbreaks, forest degradation, reduced soil fertility
Flooding	Mortality of aerobes, increase in anaerobes, restructuring of microbial community	Decreased digestive activity, reduced survival	Disruption of decomposition, enhanced wood decay, spread of diseases
Pollution (heavy metals, pesticides)	Reduced diversity, increase in resistant forms, suppression of beneficial microbes	Decline in detoxification and nitrogen fixation, increased susceptibility to pathogens, dysbiosis	Decline in forest health, intensified epiphytotics, altered soil composition, bioremediation
Changes in flora and fauna	Emergence of new associations, horizontal gene transfer among microbes	Microbiota plasticity, enhanced invasiveness, adaptation to new resources	Spread of invasive species, disruption of biocenoses, emergence of new pathogens
Disruption of symbiotic relationships	Reduction in synergy among bacteria, fungi, and protists	Impaired digestion and immunity, increased vulnerability to parasites	Decreased decomposition efficiency, impaired ecosystem recovery after disturbances/fires
Combined influence	Synergistic dysbiosis, loss of most key microbiota functions	Severe impairment of adaptive mechanisms, disruption of metabolic and immune processes	Catastrophic biodiversity loss, collapse of carbon and nutrient cycling

**Table 4:** Key evidence from primary studies and identified research gaps regarding the role of microbiota in xylophagous insect adaptation to environmental change.

Research Focus Area	Key Evidence from Primary Studies (Examples)	Identified Gaps and Proposed Experimental Approaches	Sources
Thermal Adaptation	In bark beetles (*Dendroctonus* spp.), a shift in gut bacterial communities under elevated temperature was correlated with improved fitness on a novel host tree. In mosquito models, heat-induced microbiome shifts (e.g., enrichment in *Bacillus*) were linked to altered host thermal tolerance.	Gap: causal evidence that microbiome shifts directly mediate insect host thermotolerance is scarce. Approach: perform microbiome transplantation experiments between heat-exposed and control insects, followed by fitness and physiological assays (e.g., critical thermal maximum, CTmax).	[102,113]
Drought and Humidity Stress	In termites, flooding events led to a restructuring of gut microbial communities and a significant loss of cellulolytic activity. For bark beetles (*Ips typographus*), drought stress in host trees is associated with an enrichment of xerotolerant fungal symbionts in beetle galleries.	Gap: understanding of how gut microbiota helps insects maintain water balance and digest drier wood is limited. Approach: manipulate humidity in mesocosms, track microbiome dynamics via metatranscriptomics, and measure insect water retention and digestive efficiency.	[115,124]
Pollution Detoxification	Gut bacteria of wood-feeding insects (e.g., *Pseudomonas*) demonstrate capabilities for heavy metal bioremediation in vitro. Insecticide-degrading genes have been identified in the gut metagenomes of various pest insects.	Gap: the in vivo contribution of these microbes to host detoxification and survival under chronic pollution is not quantified. Approach: use gnotobiotic insects colonized with specific degradative bacteria and challenge them with pollutants to measure survival, detoxification metabolite profiles, and microbiome stability.	[130,131,134]
Microbiome Transmission and Plasticity	Mycangia in ambrosia beetles maintain specific fungal symbionts across generations, yet allow for environmental acquisition. Horizontal gene transfer among gut bacteria is a proposed mechanism for rapid adaptation in longicorn beetles.	Gap: the relative contribution of vertical vs. horizontal transmission to adaptive potential under climate change is unknown for most taxa. Approach: conduct multi-generational selection experiments in controlled environments, tracking microbiome heritability and adaptive trait gain using metagenomic sequencing.	[93,113]
Host-Microbiome Coevolution	In cerambycid beetles, metatranscriptomes reveal coordinated expression of host and microbial genes in response to different host plants. Dual bacterial symbionts in a xylophagous beetle family show evolutionary stability and complementary nutritional roles.	Gap: lack of predictive models on how host genetics and microbiome composition interact to determine resilience. Approach: integrate genome-wide association studies (GWASs) of the host with microbiome profiling across populations under environmental clines to identify holobiont adaptation signatures.	[113,170]

## Data Availability

No new data were created or analyzed in this study. Data sharing is not applicable to this article.
